# Supplementary figures and images for: Mechanical control of osteoclast fusion by membrane-cortex attachment and BAR proteins
Source: J Cell Biol. 2025 May 8;224(7):e202411024. doi: 10.1083/jcb.202411024 (PMC12060795; doi:10.1083/jcb.202411024)

Fig. 3A

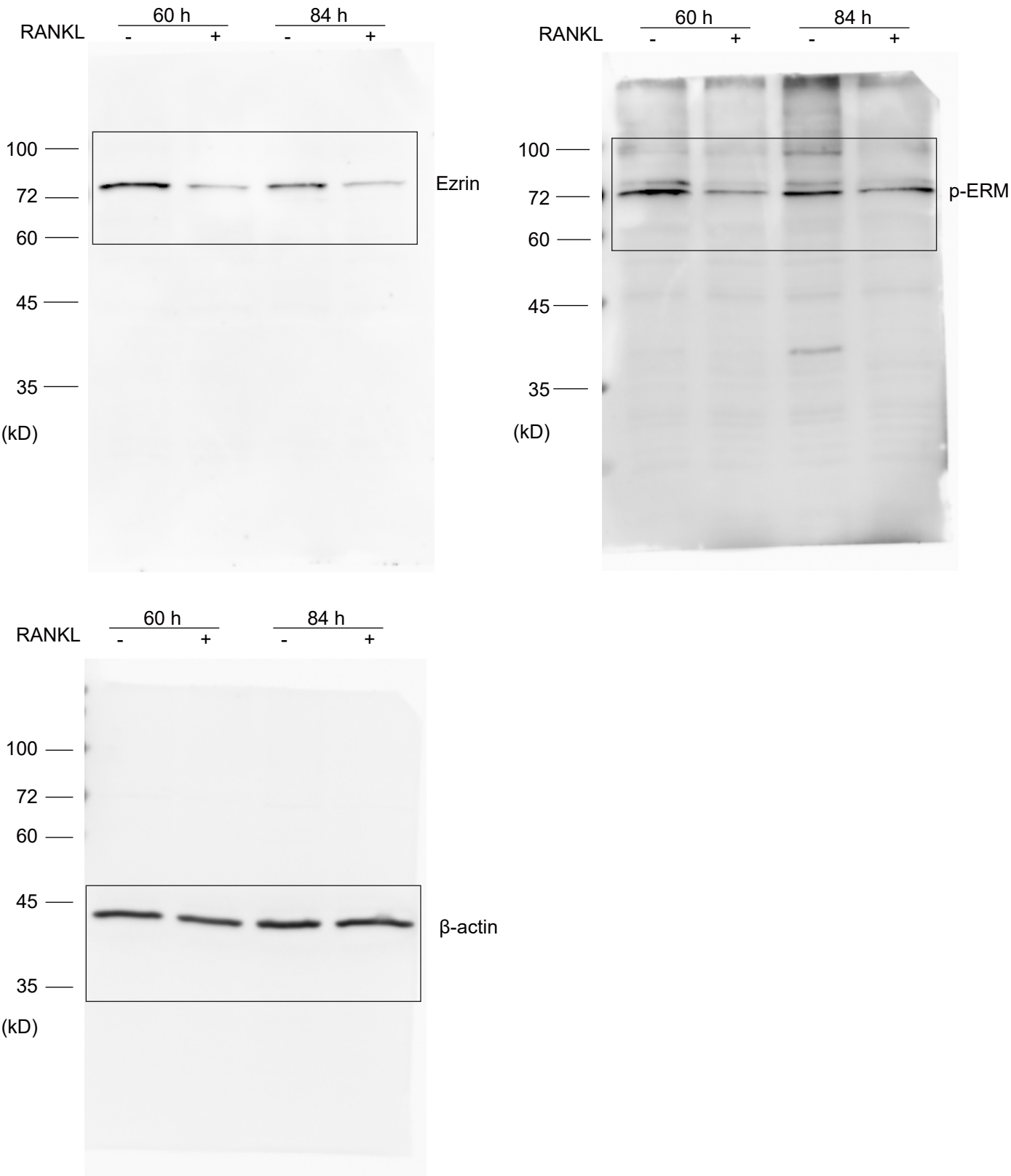

Supplement: SourceData F3 — is the source file for Fig. 3. [file jcb_202411024_sourcedataf3.pdf]

**Fig. S2A**

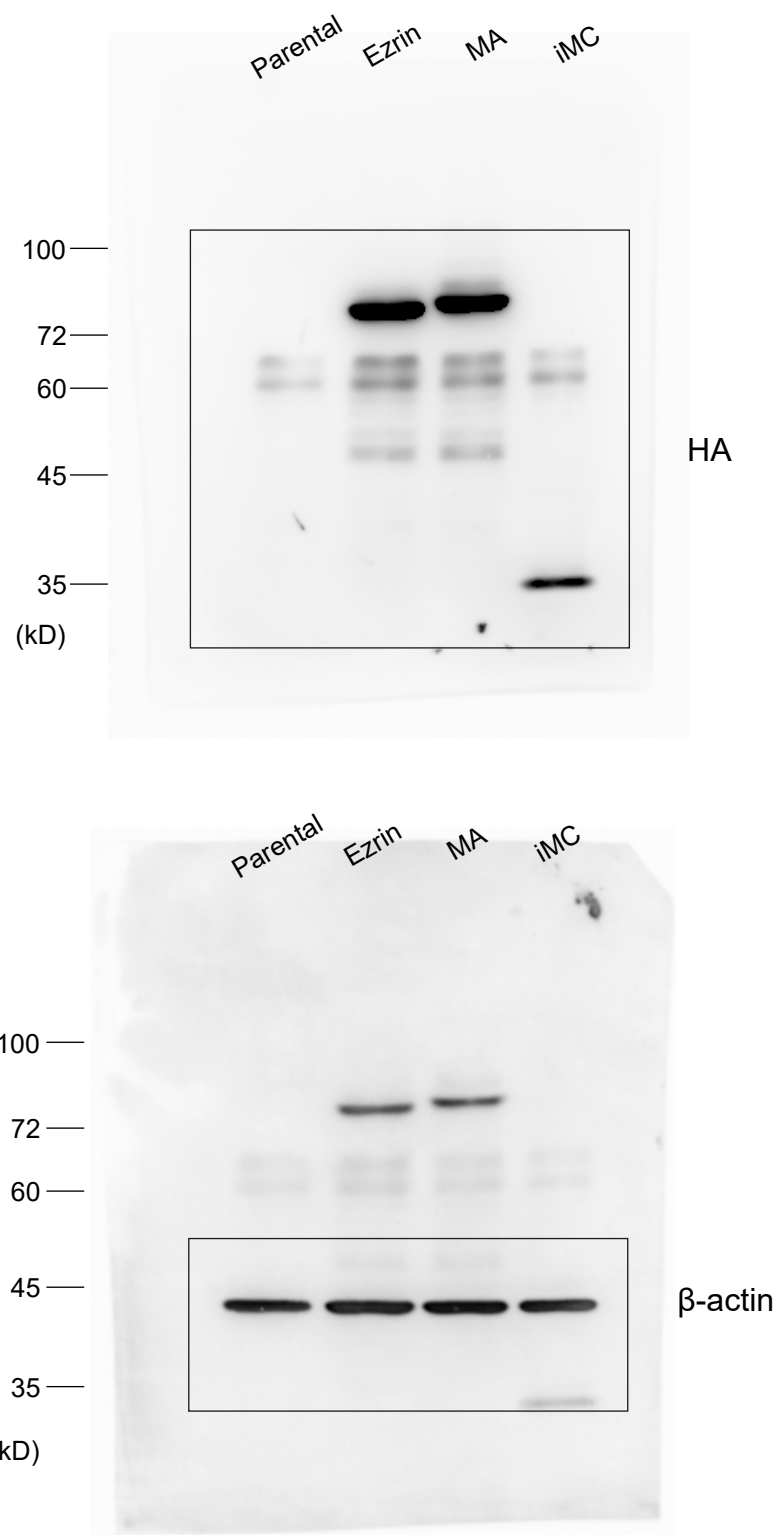

**Fig. S2B**

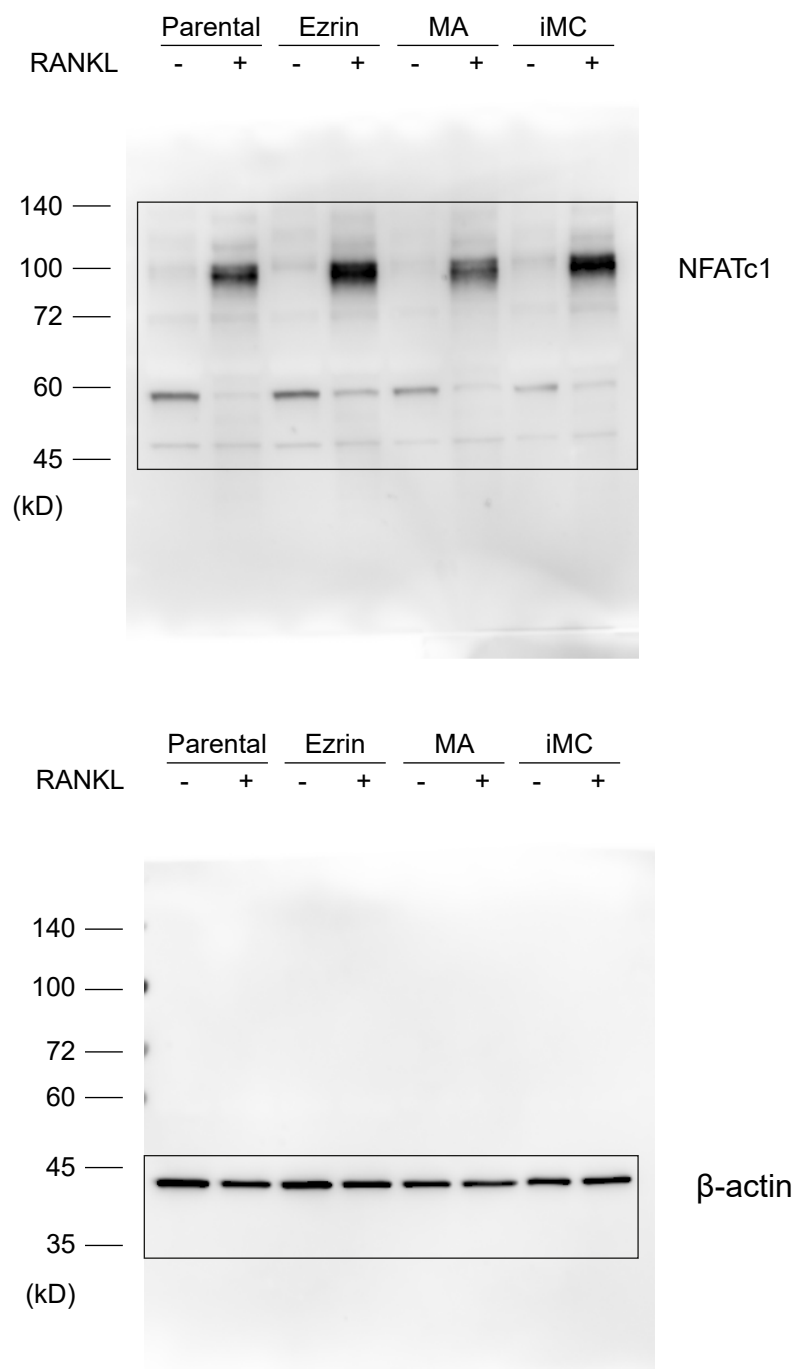

Supplement: SourceData FS2 — is the source file for Fig. S2. [file jcb_202411024_sourcedatafs2.pdf]

**Fig. S3A**

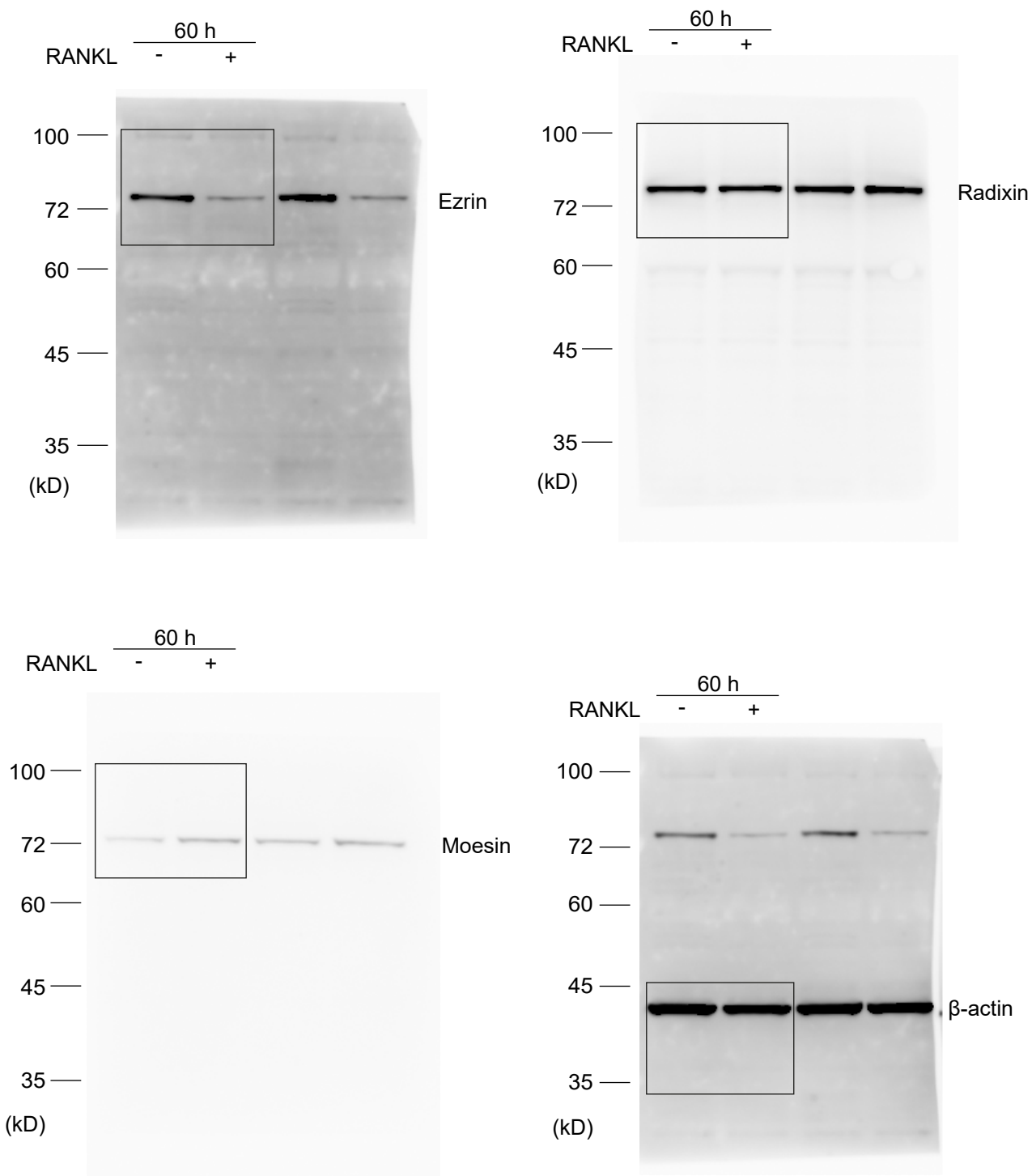

Fig. S3E

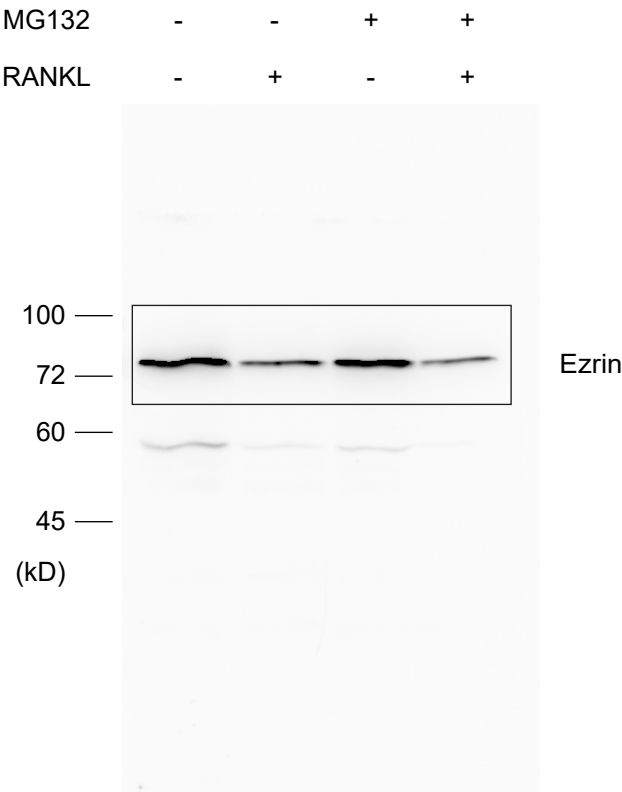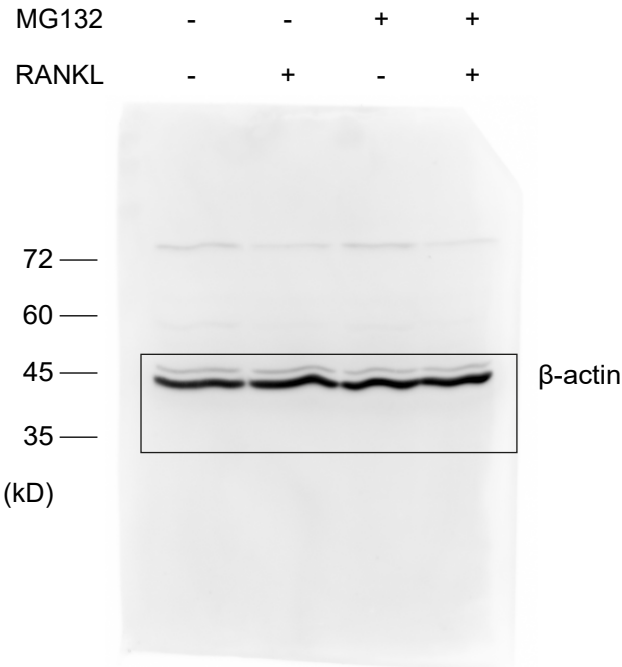

Fig. S3K

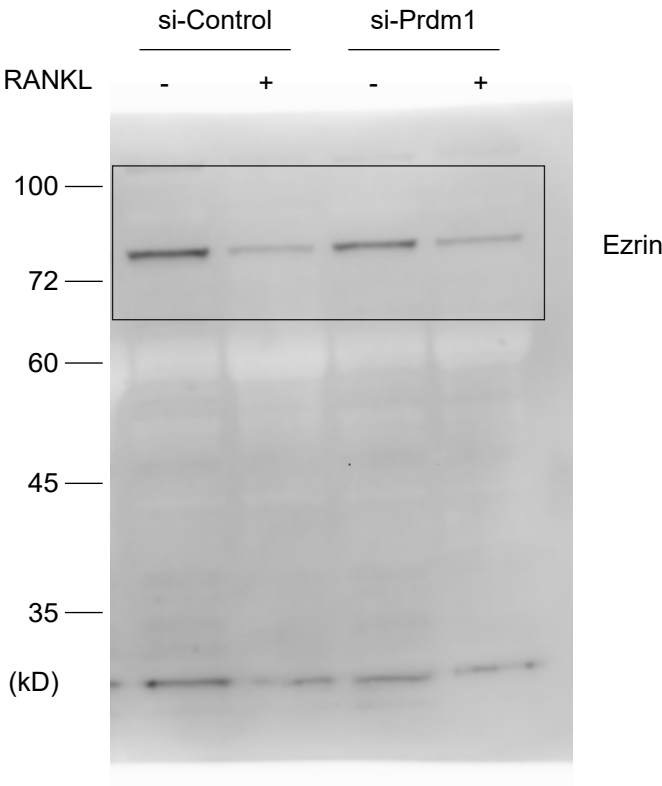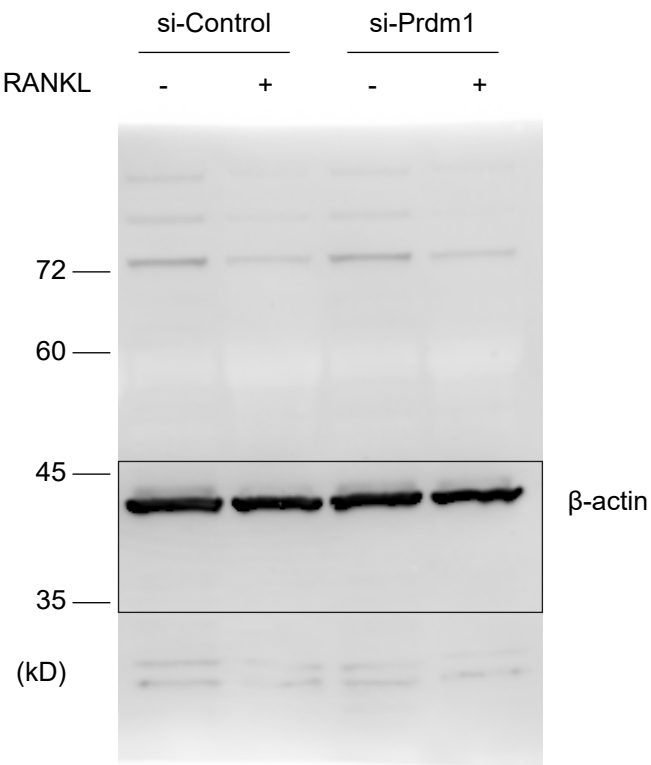

**Fig. S3M**

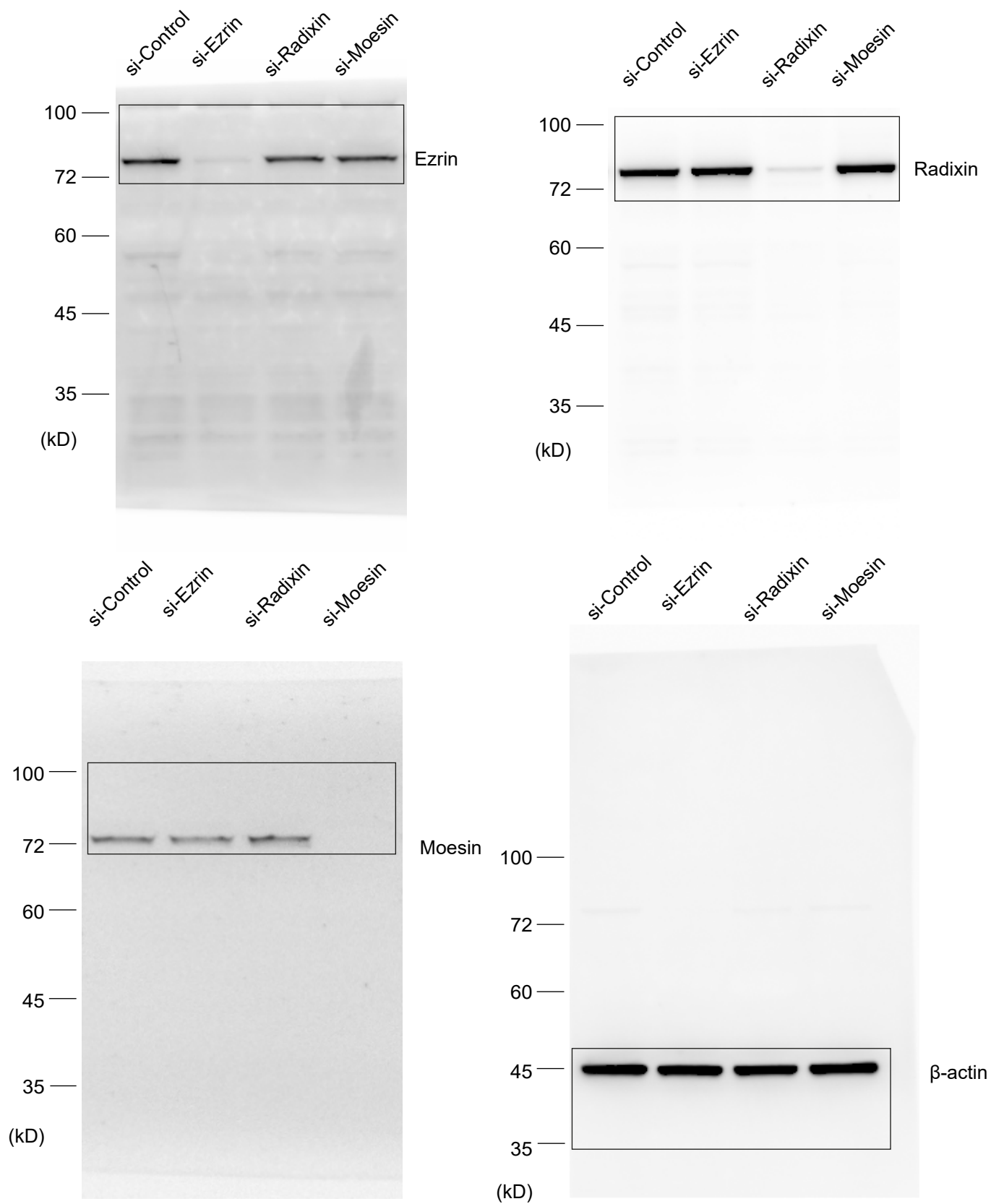

Supplement: SourceData FS3 — is the source file for Fig. S3. [file jcb_202411024_sourcedatafs3.pdf]
